# Bacterial findings in optimised sampling and characterisation of *S. aureus* in chronic rhinosinusitis

**DOI:** 10.1007/s00405-016-4239-3

**Published:** 2016-08-18

**Authors:** Ulrica Thunberg, Bo Söderquist, Svante Hugosson

**Affiliations:** 1 Faculty of Medicine and Health, School of Health and Medical Sciences, Örebro University, Örebro, Sweden; 2 Faculty of Medicine and Health, School of Medicine, Örebro University, Örebro, Sweden; 3 Department of Otorhinolaryngology, Faculty of Medicine and Health, Örebro University, SE-70182 Örebro, Sweden

**Keywords:** *Staphylococcus aureus*, Sinusitis, Nasal polyps, Sampling studies, Bacterial typing

## Abstract

The bacterial spectrum in chronic rhinosinusitis (CRS) is clinically relevant. This study aimed to compare two sampling techniques and to characterise Staphylococcus aureus isolated from CRS patients. Bacterial specimens were collected from the nares and maxillary sinus in 42 CRS patients and from the nares in 57 healthy controls. Maxillary sinus sampling was performed in two ways in each patient: with a cotton-tipped aluminium swab through the enlarged sinus ostium, and with a protected brush. *S. aureus* was characterised by DNA-sequencing of the repeat region of the *S. aureus* protein A gene, spa typing. The protected brush technique was superior to the cotton-tipped aluminium swab in reducing contamination rate. However, the two sampling methods were consistent in terms of clinically relevant bacterial findings, and the easy-to-handle cotton-tipped swab can still be recommended when culturing the maxillary sinus. Patients showed a significantly higher presence of *S. aureus* in the nares compared with healthy controls, and healthy controls showed a significantly higher presence of coagulase-negative staphylococci in the nares compared with patients. The spa types were identical for the nares and maxillary sinus in all patients except one. The sampling techniques showed equivalent results, indicating a low risk of unnecessary antibiotic treatment when using the easy-to-handle cotton-tipped aluminium swab. The high rate of identical spa types of *S. aureus* isolated from the nares and maxillary sinus of CRS patients might indicate colonisation of the maxillary sinus from the nares.

## Introduction

Chronic rhinosinusitis (CRS) affects more than 10 % of the European population [1]. The condition is characterised by nasal congestion, nasal discharge, headache, facial fullness, and changes in smell and taste lasting longer than 3 months, and is verified by nasal endoscopy and/or computerised tomography of the sinuses [2, 3]. The disease is probably multifactorial, and different factors have been suggested to affect its development, including environmental factors and host factors [4–7]. Reduced ventilation of the sinuses due to blockage of the ostiomeatal complex in the middle nasal meatus is thought to be one factor [6, 8]. Reduced oxygen pressure in the sinus and absorption of oxygen might promote bacterial growth [9]. Allergy and asthma are suggested to enhance mucosal swelling and cause obstruction of the ostium, thereby predisposing for CRS. Immune deficiencies, cell membrane sodium and chloride channel malfunction, and ciliary dysfunction may also be contributing factors [10]. Important environmental factors in development of CRS are probably microbial, especially fungi and bacteria contributing to chronic mucosal inflammation [7]. Bacterial biofilms are often found in sinuses in patients with CRS undergoing sinus surgery and are associated with more severe disease [11, 12]. Biofilms are complex structures composed of communities of microbs embedded within an extracellular matrix, predominantly polysaccharides. Bacterial involvement is well accepted in the pathogenesis of acute sinusitis, where *Streptococcus pneumoniae*, *Haemophilus influenzae*, and *Moraxella catarrhalis* are the most common bacterial findings [5, 13]. The role of bacteria in CRS is less clear, and findings of *Staphylococcus aureus*, coagulase-negative staphylococci (CoNS), and anaerobes seem to dominate to various extents in different studies [13–19]. However, a Brazilian study of 62 samples from maxillary sinuses of CRS patients found no anaerobes, and *Pseudomonas aeruginosa* was the most commonly found bacterium [20]. The variability in microbial presence in different studies might be a result of differences in culturing techniques, contamination of samples, patient selection, ethnic origin of the patients, and pre-treatment regimens. In addition, handling of samples can affect growth due to the high sensitivity of the anaerobes. *S. aureus* in maxillary sinus cultures has been reported in about 25 % of patients with CRS [21]. A meta-analysis supports the role of *S. aureus* in asthma and allergic rhinitis [22], and *S. aureus* has also been shown to have an association with inflammatory diseases, such as atopic dermatitis [23, 24]. *S. aureus* displays a wide range of virulence factors; among these, staphylococcal enterotoxins and toxic shock syndrome toxin-1 have been demonstrated to activate the immune system and affect proinflammatory cells by acting as superantigens. Some studies have suggested that chronic rhinosinusitis with nasal polyposis (CRSwNP) has a relationship with *S. aureus* infection and especially with staphylococcal enterotoxins as a modulator of the disease [6, 25–27]. The aim of this study was to investigate the bacterial spectrum patients with CRS, and especially the presence of *S. aureus*, and to characterise *S. aureus* isolated from the nares and maxillary sinus of CRS patients in comparison with samples from the nares in healthy controls. Another aim was to evaluate an optimised culturing technique for the maxillary sinus.

## Materials and methods

### Patients and controls

Forty-two patients with CRS were recruited at the Department of Otolaryngology, Örebro University Hospital, Sweden, from 2004 to 2010. Two ENT specialists (UT and SH) were responsible for the inclusion procedure. The diagnosis of CRS was based on history, clinical examination, and computed tomography scans according to the definitions and guidelines of the American Academy of Otolaryngology—Head and Neck Surgery [2]. A position paper on rhinosinusitis guidelines [28], prepared by the European Academy of Allergology and Clinical Immunology and approved by the European Rhinologic Society, [3] was published after the inclusion procedure for this study had started. However, the definition of this position paper matches that used in this study. Patients visiting the ENT outpatient clinic with CRS and an enlarged opening to the maxillary sinus due to previous surgery, and patients with CRS admitted for sinus surgery, were invited to participate. Nasal endoscopy was performed, and the presence or absence of nasal polyps was documented; thus, patients were identified as having either chronic rhinosinusitis without nasal polyposis (CRSsNP) or with nasal polyposis (CRSwNP). The inclusion procedure was not consecutive. Healthy volunteers were invited to participate in the study when visiting the Örebro travel consultation clinic. Fifty-seven controls were enrolled, and all of them did declare “no nasal polyps as an adult” and “no previous sinus surgery”. All participants were >18 years. There was 17/42 (40.5 %) male in the study group and 26/57 (45.6 %) male in the control group. The mean age was 52.5 years in the patient group and 50.0 years in the control group. Additional informed consent was obtained from all individual participants from whom identifying information is included in this study.

### Samples

**Fig. 1:**
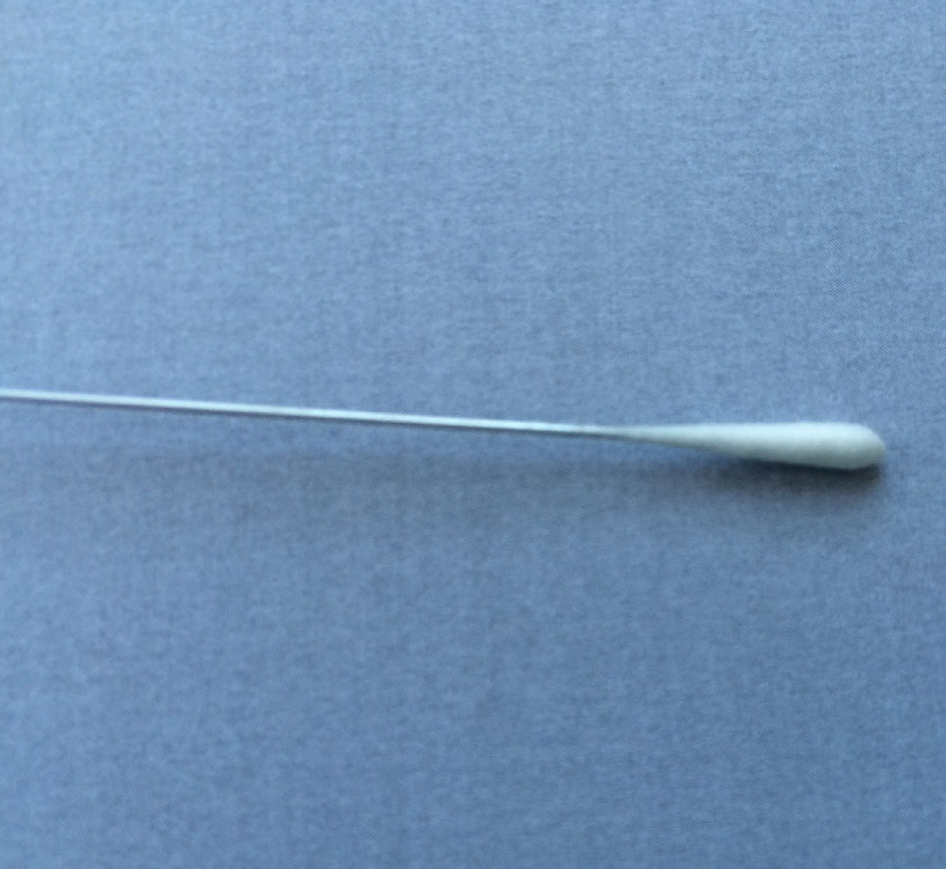
Cotton-tipped aluminium swab (Copan, Brescia, Italy)

**Fig. 2:**
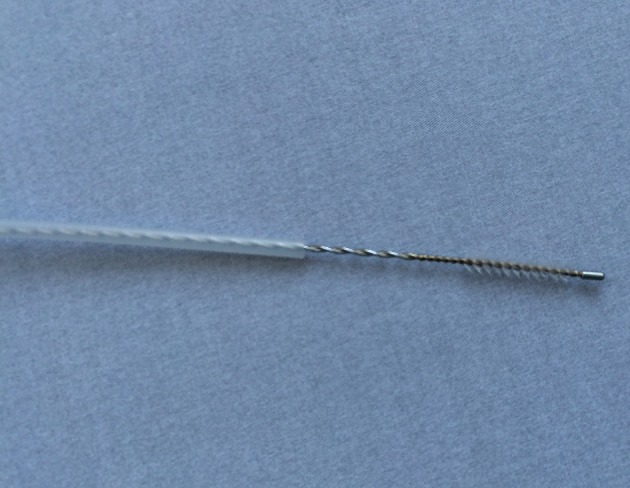
Protected brush (Olympus, model no BC-202D-3010)

Specimens were collected from the nares and maxillary sinuses of the patients, and from the nares of the healthy controls. Sampling from the maxillary sinuses of CRS patients was performed with an endoscope, using a cotton-tipped aluminium swab (Copan, Brescia, Italy) passing the nasal cavity and placed into the sinus through the enlarged sinus ostium under visual control (Fig. 1). Care was taken to avoid contamination via contact with the nose wall. Samples were also taken from the maxillary sinuses of patients with a protected brush (Olympus, model no BC-202D-3010), which could be shielded with a cover when passing through the narrow space such as the nasal cavity. This brush is often used when collecting bronchial specimen (Fig. 2). The brush was used thought an enlarged sinus ostium. Nasal specimens were collected from patients and controls using a nasal swab touching the nares. The criteria for a concordance between the two different sampling methods were defined as equal bacterial findings regarding both type and number of bacterial species.

### Microbiological analysis

The culture and species verification of bacteria was performed in accordance with routine diagnostic procedures at the Department of Laboratory Medicine, Clinical Microbiology, Örebro University Hospital. Samples were cultured on blood agar medium [4.25 % Columbia II Agar (BBL, Becton–Dickinson, Baltimore, MD, USA), 0.3 % Agar No. 2 (Lab M Ltd., Bury, UK), and 5 % bovine blood] in an anaerobic atmosphere on FAA plates (4.6 % LAB 90 Fastidious Anaerobe Agar, LAB M, Lancashire, UK) supplemented with 5 % horse blood and incubated for 2 days at 37 °C. *S. aureus* isolates were dissolved in preservation medium [yeast extract (DIFCO Laboratories, Sparks, MD, USA) and horse serum added trypticase soy broth (BBL, Sparks, MD, USA)] and stored at −70 °C pending further *spa* typing that was performed as previously described [29].

### Spa typing

Single locus DNA-sequencing of the repeat region of the *Staphylococcus protein A* gene (*spa*) for typing of *S. aureus* was performed as previously described [30].

### Statistics

Fisher’s exact test was used to calculate *p* values reflecting the difference between groups containing categorical data, and the Chi-squared test was used for the same purpose with quantitative data. A *p* value <0.05 was considered statistically significant.

## Results

Initial enrolment comprised 43 patients with CRS and 58 healthy controls. One patient was excluded due to having titanic dental implants reaching the maxillary sinus, and one of the controls was excluded due to an age below 18 years. The study group therefore consisted of 42 patients and 57 controls. Mean age was almost equal between the groups (51.5 and 50.0 years). The patient group was 46.0 % male, and the control group was 40.5 % male. Polyposis was present in 24 of the 42 (57 %) patients and none of the controls. All samples collected from the nares showed bacterial growth, though one sample in the patient group was missing. Fourteen different bacteria were identified. Table 1 shows the bacterial findings in patients and controls. The most common aerobic bacteria were CoNS, isolated from the maxillary sinus in 18/42 (43 %) patients. CoNS was found in nares in 17/42 (40 %) patients, and in 43/57 (75 %) controls (*p* = 0.0008). *S. aureus* was found in both nares and maxillary sinus in 15/42 (36 %) patients using the protected brush technique and in 18/42 (43 %) patients using the cotton-tipped aluminium swab technique (*p* = 0.66). *S. aureus* was isolated in the nares in 24/42 (57 %) patients and 16/57 (28 %) controls (*p* = 0.004). *S. aureus* findings in the maxillary sinus did not differ significantly between CRSwNP patients and CRSsNP patients (*p* = 0.35). Facultative anaerobic bacteria were found in five samples from nares of CRS patients and in two samples from controls. There was no significant difference between the patients and controls regarding the presence of facultative anaerobic bacteria in nares (*p* = 0.13). Maxillary sinus cultures using the protected brush technique showed two species present in 6/42 (14.3 %) patients, but none with three or more, while cultures using the conventional technique showed mixed flora in 14/42 (33 %) patients. At least one species of aerobic bacteria was cultured from 41/42 (98 %) patients, and anaerobic/facultative anaerobic bacteria was cultured from the maxillary sinus in 5/42 (12 %) patients. The nares showed mixed flora in 7/57 (12 %) controls and 14/42 (33 %) patients. Three samples (7 %) showed no growth, all collected with the brush technique from the maxillary sinus in CRS patients. In a comparison between the two sampling techniques, there was consistency in growth in 23/42 (56 %) patients. In 22/42 (52 %) of the brush samples, there was growth of only one species (Table 2). In one case, *S. pneumoniae* was found with the cotton-tipped aluminium swab and *H. influenzae* with the protected brush, and in another case, *S. aureus* was found with the cotton-tipped aluminium swab and *H. influenzae* with the protected brush. Furthermore, in two cases, α-streptococci and anaerobic Gram-positive cocci, respectively, were found with the protected brush but not with the cotton-tipped aluminium swab.

**Table 1:** Frequency of bacterial findings in samples from patients with chronic rhinosinusitis with nasal polyposis (CRSwNP), chronic rhinosinusitis without nasal polyposis (CRSsNP), and healthy controls

Agent identified	Maxillary sinus culture (CRS)			Nares culture (CRS)			Nares culture (controls)
	CRSwNP (*n* = 24)	CRSsNP (*n* = 18)	Total (*n* = 42)	CRSwNP* (*n* = 24)	CRSsNP (*n* = 18)	Total (*n* = 42)	Controls (*n* = 57)
Aerobic bacteria							
CoNS**	9 (38 %)	9 (50 %)	18 (43 %)	10 (42 %)	7 (39 %)	17 (40 %)	43 (75 %)
*Staphylococcus aureus*	8 (33 %)	7 (39 %)	15 (36 %)	14 (58 %)	10 (56 %)	24 (57 %)	16 (28 %)
*Haemophilus influenzae*	2 (8 %)	1 (5.6 %)	3 (7.1 %)			0	1 (2 %)
Diphtheroid rods	2 (8 %)		2 (4.7 %)	2 (8 %)	2 (11 %)	4 (9.5 %)	4 (7 %)
*Pseudomonas* sp			0		1 (5.6 %)	1 (2.3 %)	0
*Moraxella* sp			0	1 (4 %)		1 (2.3 %)	0
*α*-*haemolytic streptococci*	1 (4 %)		1 (2.3 %)	1 (4 %)	1 (5.6 %)	2 (4.7 %)	1 (2 %)
*Staphylococcus lugdunensis*	1 (4 %)		1 (2.3 %)			0	0
*Micrococcus* sp			0			0	1 (2 %)
*Streptococcus pneumoniae*	1 (4 %)		1 (2.3 %)	2 (8 %)		2 (4.7 %)	0
Enterobacteriaceae***	2 (8 %)		2 (4.7 %)	3 (12.5 %)	1 (5.6 %)	4 (9.5)	1 (2 %)
*Escherichia coli*			0	1 (4 %)		1 (2.3 %)	1 (2 %)
Anaerobic							
*Propionibacterium acnes*	1 (4 %)		1 (2.3 %)			0	0
Anaerobic Gram-positive cocci	1 (4 %)		1 (2.3 %)			0	0

* One culture is missing

** Coagulase-negative staphylococci (CoNS)

*** Except* Escherichia coli*

**Table 2:** Comparison of two different sampling techniques used in 42 patients with chronic rhinosinusitis (CRS)

Case #	Maxillary sinus (Rayon-wire swab)	Maxillary sinus (Protected brush)	Concordance*	Assumed impact on the optimal choice of antibiotics treatment when based on findings with rayon wire swab
1	*Streptococcus pneumoniae*	*Haemophilus influenzae*	–	High
2	*Staphylococcus aureus* CoNS**	*Staphylococcus aureus*	–	Low
3	CoNS Diphtheroid rods	Diphtheroid rods	–	Low
4	*Staphylococcus aureus*	*Staphylococcus aureus*	+	Low
5	CoNS	CoNS	+	Low
6	*Streptococcus* *pneumoniae* *Moraxella catarrhalis*	*Streptococcus pneumoniae*	–	Low
7	*Staphylococcus aureus* *Propionibacterium acnes*	*Staphylococcus aureus*	–	Low
8	CoNS	CoNS	+	Low
9	CoNS	CoNS	+	Low
10	*Staphylococcus aureus*	*Staphylococcus aureus* CoNS	–	Low
11	*Staphylococcus aureus*	*Haemophilus influenzae*	–	Highs
12	*Staphylococcus aureus*	*Staphylococcus aureus* CoNS	–	Low
13	CoNS	CoNS	+	Low
14	*Staphylococcus aureus*	*Staphylococcus aureus*	+	Low
15	CoNS	CoNS	+	Low
16	CoNS	CoNS	+	Low
17	*Staphylococcus aureus*	*Staphylococcus aureus*	+	Low
18	CoNS *Haemophilus influenzae* *Klebsiella* sp	*Haemophilus influenzae*	–	Low
19	CoNS	CoNS	+	Low
20	*Staphylococcus aureus*	Negative	–	Low
21	CoNS *Enterobacteriaceae*	*Enterobacteriaceae*	–	Low
22	*Staphylococcus aureus* Diphtheroid rods	CoNS	–	Low
23	*Staphylococcus aureus*	*Staphylococcus aureus*	+	Low
24	CoNS	CoNS	+	Low
25	*Staphylococcus lugdunensis*	*Staphylococcus lugdunensis*	+	Low
26	*Staphylococcus aureus*	*Staphylococcus aureus*	+	Low
27	*Staphylococcus aureus*	*Staphylococcus aureus*	+	Low
28	CoNS *Propionibacterium acnes*	CoNS	–	Low
29	CoNS	CoNS	+	Low
30	*Staphylococcus aureus*	*Staphylococcus aureus*	+	Low
31	*Staphylococcus aureus*	*Staphylococcus aureus*	+	Low
32	CoNS *Streptococcus pneumoniae*	CoNS	–	High
33	CoNS	CoNS	+	Low
34	CoNS	CoNS	+	Low
35	*Staphylococcus aureus*	*Staphylococcus aureus*	+	Low
36	*Staphylococcus aureus*	*Staphylococcus aureus*	+	Low
37	*Escherichia coli*	*Propionibacterium* *acnes*	–	Highs
38	*Staphylococcus aureus*	Negative	–	Low
39	CoNS *Propionibacterium acnes*	CoNS *α*-*streptococci* anaerobic Gram-positive cocci	–	Low
40	CoNS Diphtheroid rods *Propionibacterium acnes*	Negative	–	Low
41	CoNS *Enterococcus* sp	CoNS	–	Low
42	*Staphylococcus aureus* *Enterobacteriaceae*	*Staphylococcus aureus* *Enterobacteriaceae*	+	Low

Specimens obtained from the maxillary sinus

* Concordance meaning that the same type of bacteria was found with both culturing technique

** Coagulase-negative staphylococci (CoNS)

**Fig. 3:**
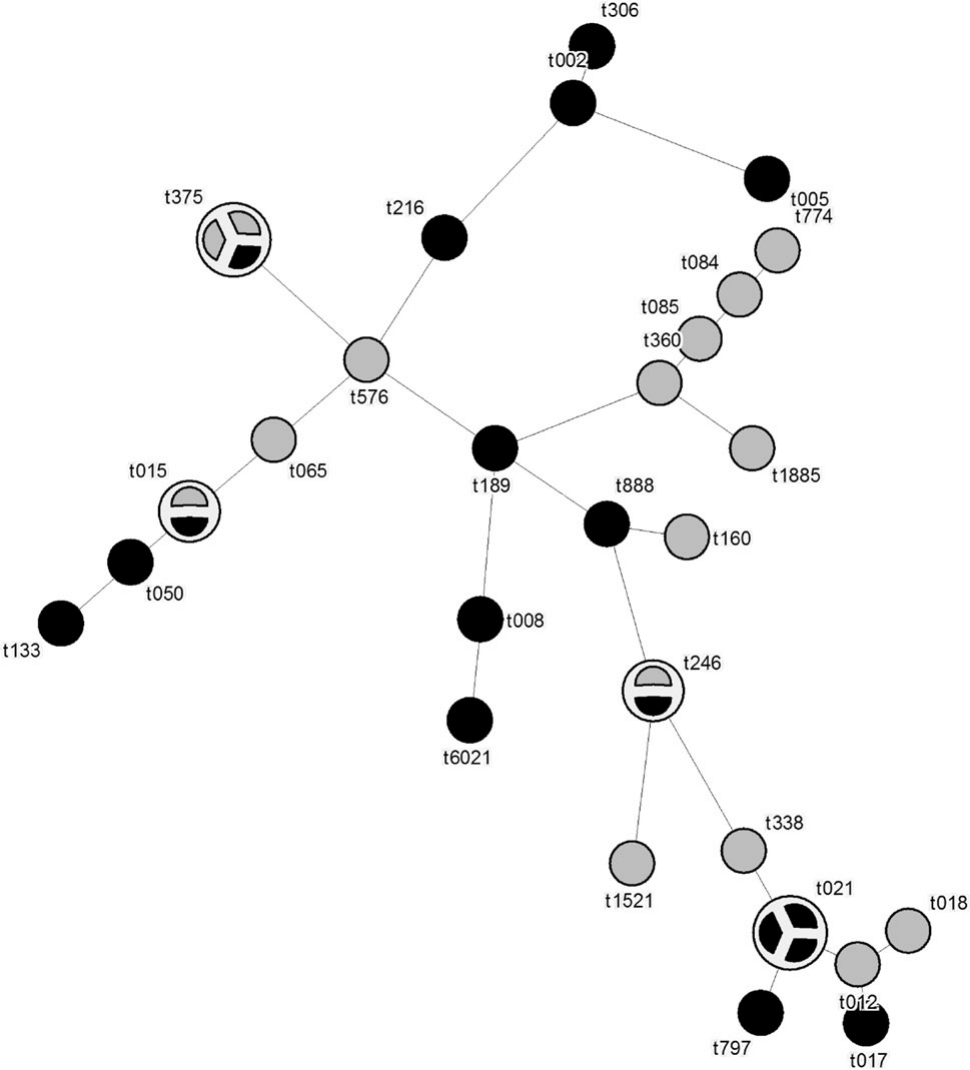
Distribution and genetic relationship of spa types of *S. aureus* isolates from the nares in controls (*grey*) and CRS patients (*black*). *One circle* represents one isolate

There was a wide variation in the distribution of *spa* types (Table 3; Fig. 3). We found identical *spa* types of *S. aureus* from the nares and from the maxillary sinus in 17/18 patients (94 %). The remaining patient with *S. aureus* isolates showed two unrelated *spa* types in the two areas: t084 and t189.

## Discussion

The isolation rate of *S. aureus* in maxillary sinus cultures from patients with CRS has been reported to be about 25 % [21]. In this study, *S. aureus* was found in 15/42 (36 %) of the maxillary sinus cultures from CRS patients with both the optimised sampling technique (protected brush) and standard cotton-tipped aluminium swab. In another three patients, *S. aureus* were found using standard cotton-tipped aluminium swab but not with the brush. This indicates a contamination rate of 3/18 (17 %) for *S. aureus*. In 17/42 (40.5 %) samples, bacterial findings with standard cotton-tipped aluminium swab differ from findings using protected brush. However, CoNS in combination with other bacterial findings using cotton-tipped aluminium swab compromised most of these differences. The protected brush technique was superior to the standard cotton-tipped aluminium swab in reducing the contamination of CoNS and *S. aureus* when culturing maxillary sinus. However, the clinical relevance of using this optimised technique as the protected brush is questionable. In almost all patients cultured with the cotton-tipped aluminium swab, this culturing technique seemed to be adequate for collecting clinically relevant samples, and thus have a low impact on antibiotic treatment (Table 3)*. S. aureus* is thought to persistently colonise the nares of about 20 % of the population (range 12–30 %), and approximately 30 % of the remainder is intermittent carriers (range 16–70 %) [31, 32]. Wertheim et al. regarded the nose as the major site of *S. aureus* carriage, and from here, the organism is thought to spread to other parts of the body [32]. *S. aureus* and CoNS were the predominant bacterial findings in the maxillary sinus of our patients with CRS. In addition, the number of patients with CRS with *S. aureus* in the nares was statistically significantly higher than the number of controls with *S. aureus* in the nares, while the number of patients with CoNS was statistically significantly lower compared to controls. The high *S. aureus* colonisation rate of the nares in patients with CRS may reflect its importance in the CRS disease. A hypothesis based on our findings might be that *S. aureus* counteracts the CoNS in the nares and promotes colonisation of *S. aureus* in the maxillary sinus through transport of bacteria across the mucus conjoining the nares with the sinus.

**Table 3:** Distribution of spa types of *S. aureus* isolates from patients with chronic rhinosinusitis (CRS) in both maxillary sinus and nares

Case #	*S. aureus* isolated in Maxillary sinus, CRS	*S. aureus* isolated in Nares, CRS
*1*	*t246*	*t246*
*2*	*t050*	*t050*
*3*	*t008*	*t008*
*4*	*t021*	*t021*
*5*	*t021*	*t021*
*6*	*t015*	*t015*
*7*	*t021*	*t021*
*8*	*t375*	*t375*
*9*	*t216*	*t216*
*10*	*t017*	*t017*
*11*	*t133*	*t133*
*12*	*t002*	*t002*
*13*	*t084*	*t189*
*14*	*t61*	*t6021*
*15*	*t797*	*t797*
*16*	*t888*	*t888*
*17*	*t005*	*t005*
*18*	*t306*	*t306*

Furthermore, identical *spa* types in both the nares and maxillary sinus were found in all patients with findings of *S. aureus* in both locations (with one exception), which supports the theory that the nares can be the primary site from where the bacteria can spread and colonise the maxillary sinus. *S. aureus* has in certain circumstances the ability to produce enterotoxins acting as superantigens that bind to T cells and exaggerate disease severity and expression [25], and cause serious invasive diseases such as sepsis with or without infective endocarditis and necrotising pneumonia as well as mild diseases, such as superficial skin and soft tissue infections. *S. aureus* enterotoxins may play a role in the severity of CRS, especially CRSwNP [27, 33, 34]. The ability of superantigens to enhance inflammatory reactions [25] might have a connection to the predominance of *S. aureus* in CRS, again especially CRSwNP [35]. An increased immune response to *S. aureus* enterotoxins has been demonstrated in nasal polyposis tissue, resulting in more pronounced eosinophilic inflammation and higher local immunoglobulin E production against staphylococcal enterotoxins in patients affected by CRSwNP [26, 36]. Our study included 23 patients with CRSwNP and 19 with CRSsNP. We could not find any differences in bacterial findings in nares or maxillary sinus when comparing these groups, but the two groups were small and comparison is difficult. Brook et al., who studied the microbiology of the maxillary sinus in 48 patients with CRS, also found no difference between CRSwNP and CRSsNP [37] which recently also was shown by Brook et al. [38]. Another study showed no significant difference in the prevalence of genes encoding virulence factors between isolates from patients with CRS and healthy controls [39]. However, a large meta-analysis by Ou et al. based on 12 case–control studies with a total of 340 cases and 178 controls demonstrated a relationship between the presence of *S. aureus* superantigens and the persistence and severity of CRSwNP [27].

The high rate of *S. aureus* in the maxillary sinus in patients with CRS in our study strengthens the importance of *S. aureus* for the pathogenesis of the disease, but its role needs to be further investigated. Larger studies could hopefully lead to valuable knowledge about the role of *S. aureus* in CRS. Sweden is a low endemic area for methicillin-resistant *S. aureus* (MRSA), and there was only one MRSA isolate in our study; this is probably also due to a generally low prescription of antibiotics in Sweden, which leads to low incidence of resistant isolates. All participants were living in Sweden, but we have no information on ethnic origin, which may have been of interest. Some patients were included in conjunction with sinus surgery, and some had a previous history of sinus surgery and were included as outpatients at the otolaryngology clinic. However, a previous study indicates that endoscopic sinus surgery does not change the bacterial flora, though it does change the presence of bacterial biofilms in the sinus [40].

## Conclusion

Contamination with CoNS was common using cotton-tipped aluminium swab technique. However, the protected brush technique did reduce the contamination rate but does not significantly improve the reliability of bacteriological diagnostics for practical clinical use. The risk of unnecessary antibiotic treatment is, therefore, thought to be low when using the easy-to-handle cotton-tipped aluminium swab. Furthermore, *S. aureus* was found in 36 % of the maxillary sinus samples from the CRS patients, and the number of patients with *S. aureus* in the nares was statistically significantly higher than the number of controls with *S. aureus* in the nares, while the number of patients with CoNS in the nares was statistically significantly lower compared to controls. These findings, together with a very high rate of identical *spa* types of *S. aureus* from patients with *S. aureus* in both nares and maxillary sinus, might indicate colonisation of the maxillary sinus from the nares and presumably a relationship between *S. aureus* and CRS.
